# Pseudo-Membranous Cynanche Trachealis, Following Scarlatina Simplex

**Published:** 1847-12

**Authors:** M. H. Fee

**Affiliations:** Hiltonsville, Indiana


Art. VI.—*Pseudo-Membranous Cynanche Trachealis, following Scarlatina Simplex.* By Dr. M. H. Fee, Hiltonsville, Indiana.

H. J., a little girl aged five years, was, about a month since, taken with scarlatina simplex, which required no medical attention, the child not being ill enough to render confinement to her room necessary. About two weeks after the first appearance of the scarlatina, and after the very slight desquamation was completed, I was called to see her. I found a congested state of the mucous membranes generally, and of the mucous membrane of the bowels particularly, the tongue being of a deep red color. I ordered minute portions of calomel with Dover's powder, to be given every two hours till six were given; afterwards to move the bowels with ol. ricin., with enough paregoric to insure the mild operation of the oil. About 24 hours after I visited her and found her perfectly relieved, and rapidly convalescing. On Friday, 19th inst., she was able to leave the room and play at large with her little brothers and sisters. The day was damp and misty. In the evening a slight swelling was discovered in the substance of the right parotid gland, but it excited no apprehension for the safety of the child. During the next day the swelling increased, and the evening showed the presence of some fever. On Sunday morning the swelling and fever had both increased to a high degree. About 10, a. m., I was sent for, but being absent, did not see her till 1, p. m. I found the parotid gland as large as the longitudinal sectional half of a goose egg; the lining membrane of the mouth, palate, fauces, pharynx, and glottis much injected and intensely red, inflamed and swollen. I ordered a solution of tartarized antimony, 1 gr. to 12 teaspoonfuls of water, of which, 1 teaspoonful to be given every hour until pretty free emesis was produced. To be aided by diluent and diaphoretic teas; a blister applied to the throat, and salts to be given in six hours if the antimonial did not move the bowels within that time. Also directed all the inflamed membrane within reach to be touched occasionally with a solution of 5 grs. nit. argenti in pure water 1 ℥f. Visited her at 9, P. M.; found the fever much moderated. She had vomited two or three times, but had had no alvine dejection; mixed and gave 2 teaspoonfuls salts, and ordered the tartar to be continued through the night at intervals of 2 or 3 hours, with frequent draughts of tea of asclepias tuberosa.

Monday, 8, A. M., found the gland reduced in size, the redness of the throat less, with slight general diaphoresis. Ordered the tartar to be continued every 3 hours, with asclepias tea, and salts sufficient to keep the bowels loose. At 3, P. M., found her sitting up in bed, with less difficulty in breathing, moist skin, good blister, and less swelling in the gland. Thought her manifestly improving; touched her throat with nit. argent., and directed the tart. antim. to be given at longer intervals. One hour after I was summoned in great haste, the messenger affirming that the child was dying. As soon as I entered the room I heard the shrill metallic cough so characteristic of cynanche trachealis. There was great difficulty of breathing, with sleepy expression of eye. The prolabia were bluish, and a white streak starting at the alæ nasi and passing entirely around the mouth; laborious action of diaphragm and abdominal muscles; with extensive swelling of the rima glottidis; pulse slow, intermitting and dragging, taking double the usual time of one systole. I immediately gave iss. gr. sulph. cupri, which, at the end of 15 minutes, had made no impression, and fearing to increase the quantity, I trusted to large draughts of tepid water. In about 20 minutes I got slight emesis, during which a portion of pseudo-membranous secretion was expelled. The brain had now become so stupefied, that I could not, even with the most powerful emetics, make any impression. I ordered the albumen of a couple of eggs to be given, to envelope and conduct without injury to the bowels, any portion of the salt of copper which might not have been thrown up during emesis. The child rapidly grew worse. The inflammatory action was rapidly spreading through the lining of the bronchi and their ramifications. The pseudo-membranous secretion was on the increase. About 10 o'clock she was nearly pulseless, and raving incoherently. Her lips were livid, face swelled, and eyes turned back under the lids. I scarified and applied a cup at the upper part of the sternum, and drew about iss ℥. of blood, with marked relief, lasting about 15 minutes, when the symptoms became still more unfavorable. Despairing of saving her by any means short of tracheotomy, I proposed the operation, but was promptly refused by the parents. Not willing to give my patient up, I determined to apply the cups every half hour; about the third application, the lips partially regained their normal color, and the pulse, which before intermitted once in every three beats, now intermitted but once in ten. Seeing that the impression from the cups was not likely to be permanent, I concluded to make an eschar at the upper part of the sternum with nitric acid. The relief was immediate. The cups were applied once afterwards. Perfect sensibility and consciousness shortly returned; at which time I gave an emetic of tartarized antimony, which operated freely, and expelled a piece of membrane four inches in length. By morning she was expectorating freely, but as the day advanced her cough became rather croupy, for which I ordered the tart. solution to be continued every hour till I should visit her in the evening, with a tablespoonful of mucilage of gum arabic occasionally. At my evening visit she was again expectorating moderately. Put her on the use of infusion senega, with tart. antimony and carb. soda, under which she rapidly convalesced, and is now, 27th, in good health, the parotid gland having regained its normal size.

From this case it appears—

1st. Inflammations in the neighborhood of the pharynx, in children, should be held as possible originators of croup.

2d. That the sequelæ of scarlatina simplex are generally more to be dreaded than those of the worse forms of scarlatina. These conclusions are drawn from my own observation in my practice in scarlatina this summer. I have had no sequel to follow the anginose variety in any case, and but once in the malignant variety, while in at least 25 per cent. of the simple variety complications occurred, generally in the form of frontal abscess, chronic swelling of the parotids, and lower extremities, rheumatic pains of the joints, &c., &c.

3. That emetics are of no service after the brain has measurably lost its sensibility from want of decarbonization of the blood.

4th. That cupping, unaided by more powerful revulsives, is of but little service, because it does not create sufficient irritation to keep up a permanent afflux of blood to the part cupped.

5th. And lastly: That it is a culpable waste of time to trust to vesication, with any thing slower in its operation than boiling water or aquafortis.

September 27, 1847.

